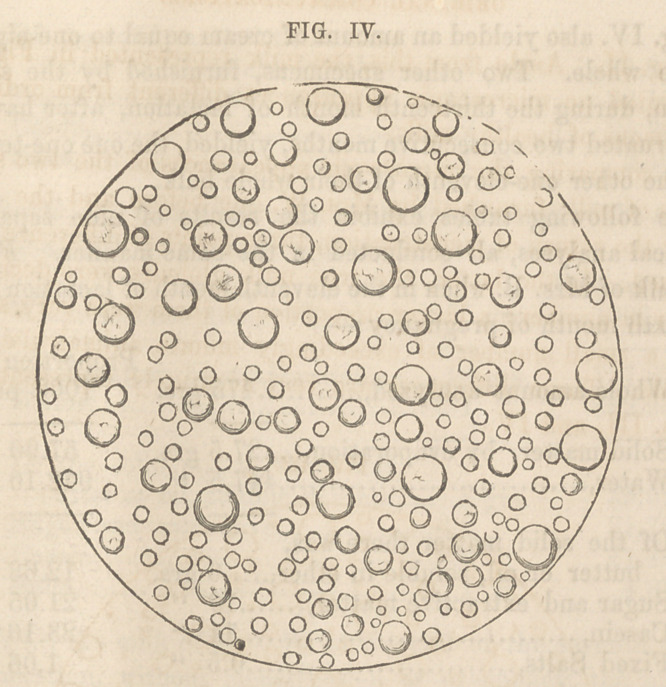# Report on the Changes in the Composition and Properties of the Milk of the Human Female, Produced by Menstruation and Pregnancy

**Published:** 1856-12

**Authors:** N. S. Davis


### THE NORTH-WESTERN

### MEDICAL AND SURGICAL JOURNAL.

(NEW SERIES.)

VOL. V. DECEMBER, 1856. NO. 12.

### ORIGINAL COMMUNICATIONS.

ARTICLE I.—*Report on the Changes in the Composition and Properties of the Milk of the Human Female, produced by Menstruation and Pregnancy; read before the American Medical Asssociation, at its Annual Meeting in May*, 1856. By N. S. DAVIS, M.D. &c.

At the Annual Meeting of this Association in May, 1855, I had the pleasure of submitting a brief report on the best methods of preserving milk, in a state of freshness and purity, during long periods of time; and I now propose to occupy your attention with such facts and observations as I have been able to collect concerning the other important topics referred to your Committee for investigation.

The opinion has long been prevalent, both in and out of the profession, that the milk of mothers, who have become again pregnant during the period of lactation, is not capable of affording a sufficient amount of healthy nutritive matter for the proper nutrition and development of the nursing infant. The number of recorded observations calculated to form a basis for this opinion, is very small; and, so far as I have been able to learn, no attempts have been made, either by microscopic or chemical examinations, to ascertain the precise changes, if any, which take place in the milk in consequence of the supervention of pregnancy or menstruation. In Hassell's work on *Microscopic Anatomy*, we find only the following paragraph:—"The milk of women, in whom the natural periods have returned during the course of lactation, has likewise been carefully examined. Except in a single instance, however, it has not been found to present anything remarkable in its characters. In the case referred to, it had degenerated to the condition of colostrum, and contained the granular colostrum corpuscles."

Lehmann, in his work on *Chemical Physiology*, just issued from the press in this country, says, in speaking of the morbid changes in milk, that "Epithelial cells, mucous corpuscles, fibrinous clots, blood corpuscles, infusoria (vibrio bacillus,) and byssus (blue milk) are rare admixtures, purely accidental, or caused by pathological affections of the mammary glands."

Dr. Carpenter, and other writers, allude to the subject in the same general terms.

Being unable to find already on record any number of analyses of milk secreted during either menstruation or pregnancy, I have embraced every opportunity afforded me during the past year for making analyses, both chemically and with the microscope.

Analyses of healthy human milk have been made by Simon, L'Heretier, Chevallier and Henri, Vernois, and several others; and the results obtained by them clearly establish the fact, that the relative proportion of the constituents of healthy milk varies much in different individuals, and even in the same individual at different times and under the influence of variations in diet, exercise, &c.

To obviate, as far as possible, any erroneous inferences from the occurrence of these normal variations, I have, in this report, placed in comparison only the milk of the same female, taken at different periods of time, but under circumstances in regard to diet, exercise, &c. as nearly uniform as possible. To determine the effects of pregnancy on the composition and properties of milk, I obtained from Mrs. G. an intelligent American lady, aged about twenty-seven years, when in the eighth month of lactation she was again found to be nearly three months advanced in pregnancy, an ample supply of milk for examination. At the same time, a careful record was made concerning her health and that of her nursing child. She passed through the full period of pregnancy, underwent a safe delivery; and, when in the third month of lactation, free both from menstruation and pregnancy, she again supplied me with a sufficient quantity of milk for a full examination.

For another comparison of the same kind, I obtained from Mrs. B. an Irishwoman, aged about thirty-five years, when in the eleventh month of lactation and the beginning of the sixth month of pregnancy, one specimen of milk, and another specimen after she had completed her period of gestation, and again arrived at the fourth month of healthy and uncomplicated lactation.

To determine the influence produced by menstruation, I obtained from Mrs. W. an intelligent American lady, aged twenty-five years, when in the seventh month of lactation, free from menstruation, and nursing a very healthy and well-nourished baby, an abundant supply of milk. Four months subsequently she began to menstruate and continued to do so regularly, and, at the same time, nurse her infant until the fourteenth month of lactation. During the period intervening between the second and third menstrual discharges, she furnished four more samples of milk for examination and analysis.

All the specimens of milk examined were subjected to the same processes, and with a view of determining in each:—*first*, the microscopic appearances; *second*, the relative proportion of cream which would separate by standing; *third*, the relative proportion of water and solid matter; and, *fourth*, the relative proportion of each of the following ingredients, viz.:—water, butter (or oil soluble in ether,) casein, sugar and extractive matter, and salts. To accomplish the first object, each specimen of milk, soon after its removal from the breast, was subjected to a careful examination under a good achromatic lens, magnifying one thousand diameters. The two first specimens, which were obtained from females in the state of pregnancy, presented appearances strikingly similar. A carefully-executed sketch of the field, as it appeared under the microscope, while examining the milk of Mrs. G. is herewith shown in

FIG. I.

To facilitate the comparison, I have represented (Fig. II.) the appearances of another specimen of milk, obtained from Mrs. G. after she had nursed her second child three months, and was free from the influence of either pregnancy or menstruation.

Several microscopic peculiarities were easily recognized in the specimen first examined. The globules or milk corpuscles were comparatively very few in number, and their size either very large or very small; the medium-sized corpuscles being the most deficient. Of the larger-sized corpuscles some were distinctly oval, and most of them appeared to possess double-cell walls, one within the other, precisely as described by Turpin.* A few true colostrum corpuscles were also found in both the specimens of milk from the pregnant females. Only one of them was present in the field represented on Fig. I. There was also observed, in these specimens of milk, a greater number of

* Annales des Sciences Naturelles.

FIG. II.

exceedingly minute globules of low refracting power, which are supposed to be composed of casein by M. M. Quevenne and Donne. In the plates they are mostly represented as small black spots.

Another highly-interesting peculiarity, noticed in the milk of Mrs. G. while in a state of pregnancy, was the presence of a considerable number of exceedingly small anamalculæ. Some of them appeared to be perfectly linear, like vibrios; while others were evidently enlarged at one end, somewhat resembling the human spermatozœ, though much smaller and the tail less filiform. They were all capable of an independent, though vibratile or wriggling motion, by which they were sometimes seen to move from one-fourth to one-half of the distance across the field. After repeated and careful observations, I am satisfied that the motions here described were wholly independent of any accidental motion in the fluid under examination. These apparent animalculæ are very correctly represented in Fig. I. They were also visible, though fewer in number and smaller, in the milk of the same woman when not pregnant, as represented in Fig. II. Aside from this, the milk represented in Fig. II. presented no microscopic appearances different from ordinary specimens of healthy milk.

In comparing the microscopic appearances of the two specimens of milk furnished by Mrs. W. one before and the other after the return of menstruation, three points of difference were noticed. In the latter, the true milk globules were decidedly fewer in number; a larger proportion of them were very small; and a small number of exceedingly minute animalculæ were visible. These differences are very accurately represented in Figs. III. and IV.

FIG. III.

FIG. IV.

To ascertain the relative proportion of cream which would separate spontaneously, a certain quantity of each specimen of milk was allowed to stand in an open tube thirty-six hours, when the thickness of the stratum of cream was readily determined by measurement. The milk of Mrs. G. obtained when she was in the eighth month of lactation and the third month of pregnancy, exhibited a quantity of cream equal to one-sixteenth part of the whole bulk of milk. That obtained from Mrs. B. when in the eleventh month of lactation and the sixth month of pregnancy, exhibited cream equal only to one-twentieth of the whole quantity of milk. The milk received from Mrs. G. during the third month of the second lactation, yielded a proportionate bulk of cream fully equal to one-eighth; and that obtained from Mrs. B. during the fourth month of the second lactation, gave one-twelfth.

These are striking differences, and well worth careful consideration.

The milk furnished by Mrs. W. when in the seventh month of healthy and uncomplicated lactation, the same as represented in Fig. IV. also yielded an amount of cream equal to one-eighth of the whole. Two other specimens, furnished by the same woman, during the thirteenth month of lactation, after having menstruated two consecutive months, yielded, the one one-tenth, and the other one-eleventh of their whole bulk.

The following tables exhibit the results of nine separate chemical analyses, all conducted in the same manner. *First*, the milk of Mrs. B. when in the eleventh month of lactation and the sixth month of pregnancy:—

| | | Proportion in 1000 parts. |
|---|---|---|
| Whole amount analyzed, | 475 grs. | |
| Solid matter, by evaporation, | 27.5 grs. | 57.90 |
| Water, | 447.5 " | 942.10 |
| Of the solid matter there was, butter or oil, soluble in ether, | 6 grs. | 12.63 |
| Sugar and extractive matter, | 10 " | 21.05 |
| Casein, | 11 " | 23.16 |
| Fixed Salts, | 0.5 " | 1.06 |

*Second*, the milk of Mrs. B. when in the fourth month of her second lactation, uncomplicated by pregnancy:—

| | | Proportion in 1000 parts. |
|---|---|---|
| Whole amount analyzed, | 829 grs. | |
| Solid matter, by evaporation, | 94 grs. | 113.39 |
| Water, | 735 " | 886.61 |
| Of the solid matter there was, butter or oil, soluble in ether, | 19 grs. | 22.92 |
| Sugar and extractive, | 24 " | 28.50 |
| Casein, | 48 " | 57.90 |
| Salts, | 3 " | 4.07 |

*Third*, the milk of Mrs. G. in the eighth month of lactation, and the third of pregnancy:—

| | | Proportion in 1000 parts. |
|---|---|---|
| Whole amount analyzed, | 475 grs. | |
| Solid matter, by evaporation, | 32 grs. | 67.36 |
| Water, | 443 " | 932.64 |

| | | |
|---|---|---|
| Of the solid matter there was, | | |
| butter or oil, soluble in ether,......8 | grs. | 16.84 |
| Sugar and extractive matter,.......11 | " | 22.10 |
| Casein, ...............................12 | " | 25.26 |
| Salts,......................................1 | " | 2.10 |

*Fourth*, the milk of Mrs. G. in the third month of the second lactation, without pregnancy or menstruation:—

| | | |
|---|---|---|
| | | Proportion in |
| Whole amount analyzed,...........205 | grs. | 1000 parts. |
| Solid matter, by evaporation,.......24 | grs. | 117.07 |
| Water, ...............................181 | " | 882.93 |
| Of the solid matter there was, | | |
| butter or oil, soluble in ether,......8 | grs. | 39.02 |
| Sugar and extractive,...............6.5 | " | 31.70 |
| Casein, ...............................8.5 | " | 41.46 |
| Salts,......................................1 | " | 4.87 |

*Fifth*, the milk of Mrs. W. at the end of the seventh month of lactation, without menstruation or pregnancy, the nursing child being very robust and healthy:—

| | | |
|---|---|---|
| | | Proportion in |
| Whole amount analyzed,.........1500 | grs. | 1000 parts. |
| Solid matter by evaporation,......203 | grs. | 135.34 |
| Water,...............................1297 | " | 864.66 |
| Of the solid matter there was, | | |
| butter or oil, soluble in ether,....67 | grs. | 44.67 |
| Sugar and extractive,................55 | " | 36.66 |
| Casein,...................................75 | " | 50.00 |
| Salts, .....................................6 | " | 4.00 |

*Sixth*, the milk of Mrs. W. in the thirteenth month of lactation and the third month of menstruation. The table presents the average results of four separate analyses:—

| | | |
|---|---|---|
| | | Proportion in |
| Whole amount analyzed,.........1467 | grs. | 1000 parts. |
| Solid matter, by evaporation,.....137 | grs. | 93.38 |
| Water,...............................1330 | " | 906.62 |

Of the solid matter there was,

| | | |
|---|---|---|
| butter or oil, soluble in ether,...44 grs. | | 29.99 |
| Sugar and extractive,................41 | " | 27.94 |
| Casein,..................................48 | " | 32.71 |
| Salts,.......................................4 | " | 2.72 |

If we may deduce conclusions from the limited number of observations and analyses here detailed, we may find a very definite answer to the question under consideration, so far as it relates to the changes in the *composition* of the milk.

*First*—The occurrence of pregnancy during lactation, produces a very marked diminution of all the solid or nutritive constituents of the milk; such diminution continuing to increase as the pregnancy advances.

*Second*—In examining the separate proximate constituents, it will be observed that a much greater relative diminution takes place in the casein, the butter or oil, and the salts, than in the sugar and extractive matter.

*Third*—There appears to be added to the milk secreted during the progress of utero-gestation, some of the *granular* bodies or colostrum corpuscles, and numerous minute infusoria or animalcular germs, which have been very rarely found in healthy milk.

*Fourth*—Changes, the same in kind, take place in the milk secreted after the establishment of regular menstruation, but much less in degree; and the relative diminution of the several constituents is more uniform.

CHANGES IN THE QUALITIES OF THE MILK.

These may be inferred, partly, from the previously ascertained changes in its composition, and partly from a direct observation of its effects on the nursing child.

From the comparatively small quantity of solid or nutritive matter in the milk secreted after the commencement of pregnancy, it is evident that it is much less capable of furnishing to the nursing child a sufficient quantity of nutritive material for the healthy development of all its tissues; while the presence of granular or colostrum corpuscles, with or without infusoria, would greatly tend to establish irritation in the mucous membranes, manifested by frequent attacks of diarrhœa, more or less emaciation, and almost constant peevishness. These inferences, drawn from a knowledge of the changed composition of the milk, are, to a certain extent at least, confirmed by direct clinical observations. Thus, the child of Mrs. G. which was only about four months old when the mother again became pregnant, was perfectly healthy and well nourished up to that time. Soon after, it began to be peevish and restless, with flatulency, and occasionally green discharges from the bowels. These changes were at first slight, but they gradually increased, and at the end of six weeks it was evident that the child had ceased to be well nourished. Its tissues had become soft and flabby, and the mucous surfaces irritable, as manifested by occasional vomiting and more frequent intestinal discharges of a green color and sometimes mixed with mucous. During the succeeding two months, the child was subjected to several attacks of severe watery diarrhœa, accompanied by so much emaciation that the parents took it from the city into the country, with the hope that a change of air would restore it. While absent, however, it was attacked with cholera morbus, and died in two or three days. To enable the reader to judge how far the period of primary dentition and the season of the year influenced the child, it is proper to state, that at no time could I discover any evidence that the advancing teeth produced irritation, either local or general. The mother became pregnant the last week in April; and the nutrition and general health of the nursing child became decidedly impaired before the end of the June following. This was too early for the influence of the season to be felt unfavorably; although the two following months (July and August) undoubtedly increased the tendency to intestinal irritation, and probably hastened the fatal termination.

The child of Mrs. B. was also healthy and well nourished until it was five months old, when the mother again became pregnant, which was in the month of March, 1855. During the month of May, the mother applied to me for advice, saying, that, for three or four weeks, her child had been unusually fretful and restless; that it was troubled with flatulency; often rejected its milk by vomiting, and was "getting poor." I examined the child's mouth, but found no swelling of the gums or other indications of irritation from teething. Although temporarily relieved by medicine from time to time, the child continued to fail in its nutrition and to become more and more subject to diarrhœa and vomiting, until during the latter part of summer it presented the appearance of extreme emaciation and anœmia. The mother persisted in nursing it until the middle of September. Soon after it was taken from the breast, it began to gain in flesh and strength, and continued to do so for two months. At the end of this time it was attacked with symptoms of sub-acute meningial inflammation, and died in about six days with evident effusion of serum on the brain.

I have been thus particular in stating the health of the children, as well as the season of the year when the mothers became pregnant, in connection with the results of the analyses and microscopic examinations of the milk, that we might have all the circumstances which could be supposed to exert an influence on the results. Since their second confinement, both mothers have enjoyed good health; and at the time they furnished the last specimens of milk for examination, the one in the third, (see Fig. 2) the other in the fourth month of lactation, their nursing children were perfectly healthy and well nourished. Since this subject has been under investigation, I have met with four other females who had become pregnant while nursing. In all but one the children began to exhibit symptoms of imperfect digestion and nutrition within two months from the time the mothers became pregnant, and continued to do so until they were removed from the breast. One of these mothers came to me for advice about the propriety of *weaning* her child in November last. She said the child had not "*grown well* for several months;" that it was exceedingly fretful and restless, with frequent disorder of the bowels. I learned that she was then in the sixth month of pregnancy, and the secretion of milk in her breasts had been insufficient for the child without the daily use of other milk. She agreed to procure for me a vial of milk from her breasts the next day, and then immediately wean the child. To her surprise, however, she found on making the trial next day that she had no milk in her breasts, being able to procure the discharge of only a very few drops of a watery fluid. The child was not put again to the breasts, and no more milk was secreted until after her subsequent confinement. Here was a case in which the progress of utero-gestation seemed to cause the entire cessation of the secretion of the mammary glands; the woman being in the mean time in robust health. The child subsequently became healthy, and remains so at the present time. On the other hand, one of the four mothers, to whom I have just alluded, became pregnant about the sixth month of lactation. The nursing child continued to enjoy good health, and its nutrition to remain nearly as perfect as before. The mother herself, however, soon began to show signs of anœmia, which increased so rapidly, coupled with much nervous irritability and tenderness of the mucous lining of the mouth, that she was compelled to wean her child at the end of the fifth month after the commencement of pregnancy. So far as I have been able to observe, the effects of menstruation, both on the quality of the milk and the health of the nursing child, are much less marked than than those of pregnancy. The child of Mrs. W. maintained good health and was well nourished throughout the whole period of nursing. During the last two months, however, after the menses had returned, it became habitually more irritable, and seemed to require additional nourishment.

Investigations, such as I have entered upon, for elucidating the questions propounded to me by the Association, require much time and labor, though the results may be stated in a few words or figures. The important practical bearing of the results obtained thus far, will be obvious to every intelligent physician. But the examinations and analyses, microscopic and chemical, should be multiplied until they are sufficient to render all conclusions, drawn from them, demonstrated truths.

## Figures and Tables

**FIG. I. f1:**
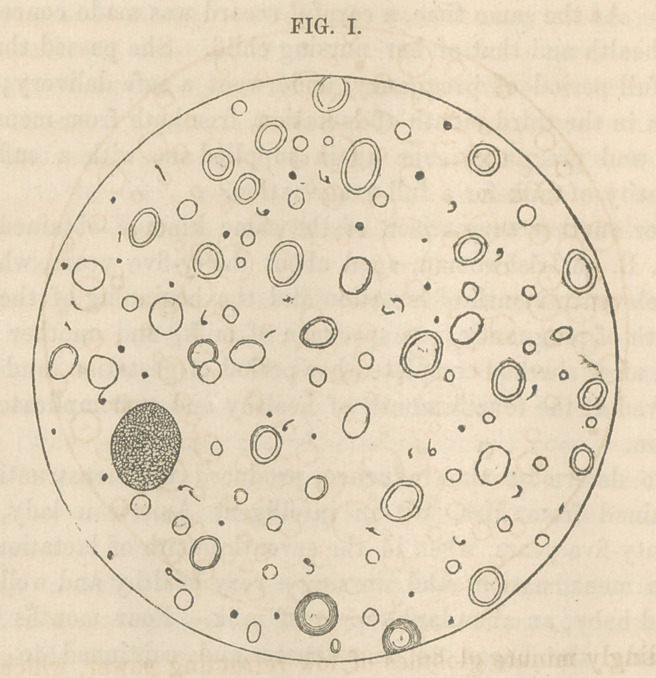


**FIG. II. f2:**
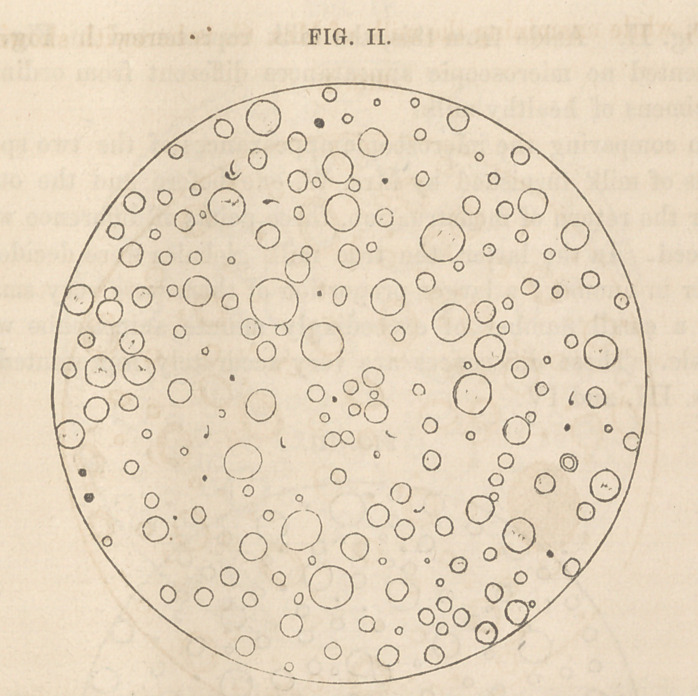


**FIG. III. f3:**
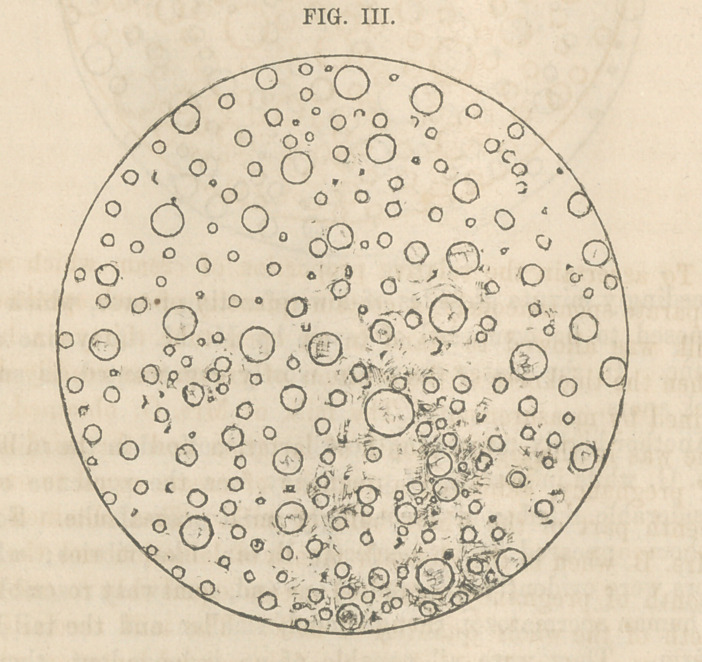


**FIG. IV. f4:**